# Sickness in Hillsborough, Ill.

**Published:** 1873-02-01

**Authors:** 


Dr. T. D. Washburn writes us from Hillsborough, Ill., that they are having considerable sickness, and more than usual fatality. Pneumonia most prevalent; also, a severe form of erysipelas; and the epidemic of influenza and catarrhal affections, which seems to follow in the course of the epizootic. Puerperal cases not doing as well as usual.

